# Asymmetry in functional connectivity of the human habenula revealed by high‐resolution cardiac‐gated resting state imaging

**DOI:** 10.1002/hbm.23194

**Published:** 2016-04-01

**Authors:** Sébastien Hétu, Yi Luo, Ignacio Saez, Kimberlee D'Ardenne, Terry Lohrenz, P. Read Montague

**Affiliations:** ^1^ Virginia Tech Carilion Research Institute Riverside Circle Roanoke Virginia 24016; ^2^ Wellcome Trust Centre for Neuroimaging University College London, 12 Queen Square London WC1N, 3BG United Kingdom; ^3^ Present address: Yi Luo is currently at Beijing Normal University 19 Xinjiekou Outer Street Haidian Beijing 100875 China; ^4^ Present address: Ignacio Saez is currently at University of California Berkeley Berkeley CA; ^5^ Present address: Kimberlee D'Ardenne is currently at Arizona State University Tempe AZ 85281

**Keywords:** substantia nigra, ventral tegmental area, fMRI

## Abstract

The habenula is a hub for cognitive and emotional signals that are relayed to the aminergic centers in the midbrain and, thus, plays an important role in goal‐oriented behaviors. Although it is well described in rodents and non‐human primates, the habenula functional network remains relatively uncharacterized in humans, partly because of the methodological challenges associated with the functional magnetic resonance imaging of small structures in the brain. Using high‐resolution cardiac‐gated resting state imaging in healthy humans and precisely identifying each participants' habenula, we show that the habenula is functionally coupled with the insula, parahippocampus, thalamus, periaqueductal grey, pons, striatum and substantia nigra/ventral tegmental area complex. Furthermore, by separately examining and comparing the functional maps from the left and right habenula, we provide the first evidence of an asymmetry in the functional connectivity of the habenula in humans. *Hum Brain Mapp 37:2602–2615, 2016*. © **2016 The Authors Human Brain Mapping Published by Wiley Periodicals, Inc**.

**Abbreviations**
BOLD: blood‐oxygen‐level dependent
DTI: diffusion tensor imaging
FA: flip angle
fMRI: functional magnetic resonance imaging
FWHM: full width at half maximum
Hb: habenula
leftHb: left habenula
PAG: periaqueductal gray
PE: prediction error
PH: parahippocampus
rightHb: right habenula
RMTg: rostromedial tegmental nucleus
ROI: region of interest
SD: standard deviation
SN: substantia nigra
TE: time to echo
TR: time to repetition
tVTA: tail of the ventral tegmental area
VTA: ventral tegmental area

## INTRODUCTION

Recently, there has been a surge of interest in the study of the habenula (Hb) network. This interest is driven largely by the habenula's proposed role in mood and addiction disorders [Bourdy and Barrot, 2012; Hikosaka, 2010; Lammel et al., 2014; Lecca et al., 2014; Proulx et al., 2014]. The Hb is an excitatory midbrain nucleus highly preserved in vertebrates [Stephenson‐Jones et al., 2012] located at the most caudal and dorsal part of the thalamus [Naidich et al., 2009]. Work on the habenula's afferents and efferents in non‐human mammalians has shown that the Hb receives inputs from several structures including the lateral hypothalamus, entopeduncular nucleus, globus pallidus, anterior cingulated, and the medial prefrontal cortex. Its outputs include the rostromedial tegmental nucleus, also called tail of the ventral tegmental area, the VTA, and serotonergic centers [Bianco and Wilson, 2009; Herkenham and Nauta, 1977, 1979; Proulx et al., 2014; Quina et al., 2015]. Because of these connections, the Hb is considered as an important relay between cortical and subcortical structures involved in cognition/emotion and reward processing. Indeed, the Hb has been shown to be involved in reward prediction error coding [Matsumoto and Hikosaka, 2007], signals important for learning and goal directed actions [Schultz et al., 1997]. Previous results have suggested that Hb activity is increased by negative reward prediction error, which inhibit the activity of dopamine neurons of the substantia nigra/VTA (SN/VTA) complex via the rostromedial tegmental nucleus [Hong et al., 2011; Jhou et al., 2009; Matsumoto and Hikosaka, 2007, 2009]. There is also strong evidence that the Hb is involved in pain processing through its connections to the limbic system [reviewed in Shelton et al., 2012a]. Although a considerable amount of data related to the connectivity of the Hb has been collected in rodents and non‐human primates [Hikosaka, 2010; Lecca et al., 2014; Proulx et al., 2014], few studies have examined its functional connectivity in humans [Erpelding et al., 2014; Hennigan et al., 2015; Ide and Li, 2011; Lawson et al., 2014; Shelton et al., 2012b].

There are known laterality differences in morphology, structure, and function in the Hb of various non‐human species [see Bianco and Wilson, 2009 for a review). However, data looking at differences between left (leftHb) and right (rightHb) habenula in humans remain sparse. Two recent fMRI studies looking at the role of the human Hb in pain processing have found conflicting results regarding laterality with one suggesting a stronger cued pain response in the leftHb [Hennigan et al., 2015] while the other found that pain versus reward coding was only different in the rightHb [Lawson et al., 2014]. Previous resting state study of Hb functional connectivity in humans did not directly test for laterality differences [Erpelding et al., 2014]. The lack of data on the Hb network and its possible asymmetry in humans can probably be explained by hurdles related to the fMRI acquisition of midbrain and diencephalon structures including their small size [Eapen et al., 2011; Lawson et al., 2013] and their proximity to important brain arteries which may affect imaging signals [Dagli et al., 1999; Enzmann and Pelc, 1992]. By using high‐resolution cardiac‐gated resting state imaging in healthy individuals, this study aims at better defining the functional coupling of the Hb and at investigating possible laterality differences in functional connectivity.

## MATERIAL AND METHODS

Fifty one healthy subjects (25 females, mean age 29 ± 11[SD]) were recruited and completed a 5‐minute resting state fMRI session during which they were instructed to remain still, clear their mind and let it wander while watching a fixation cross. Seventeen subjects were removed because of large head motion (*n* = 3) or problems with the equipment/acquisition (*n* = 14). Therefore, 34 subjects (18 females, mean age 30 ± 12[SD], min = 19, max = 58) were included in the final analyses.

**Figure 1:**
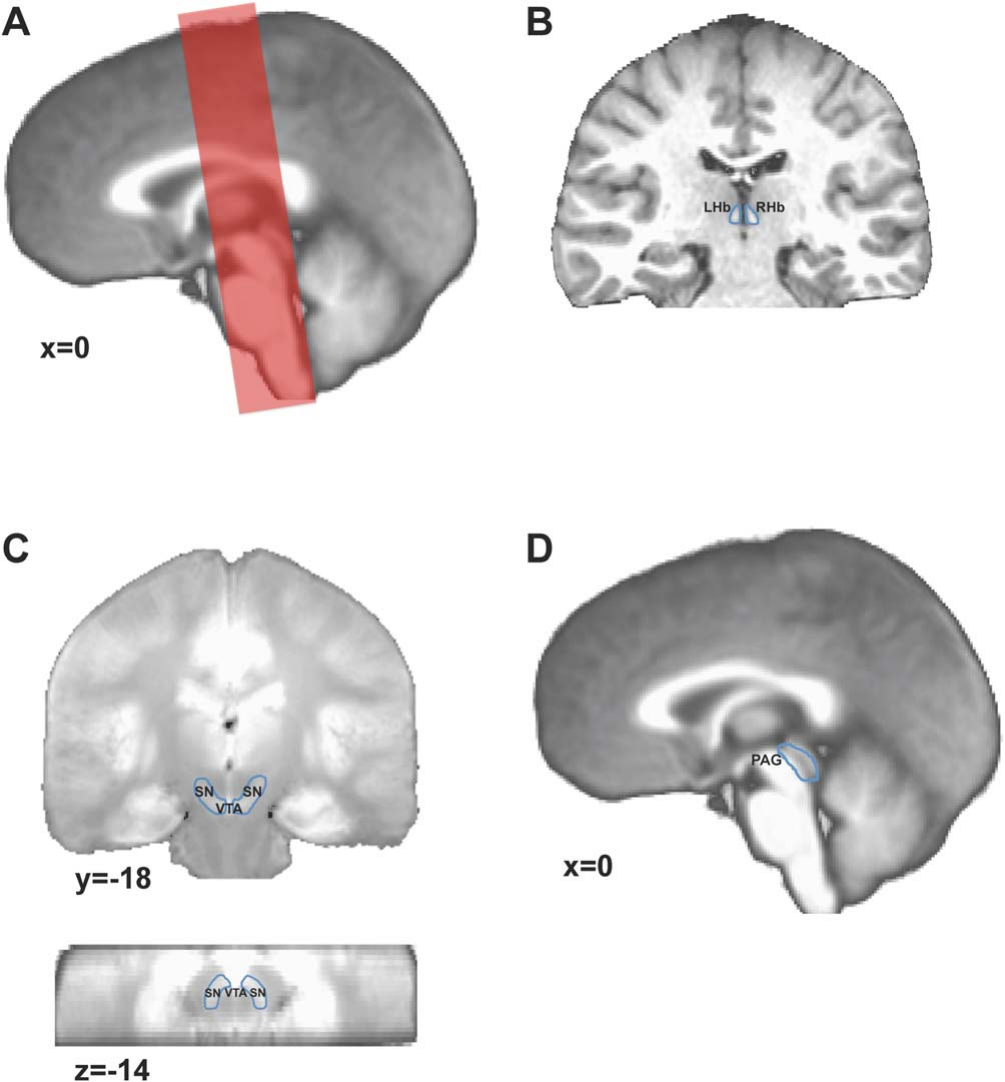
(A) Example of the coronal slab placement overlaid over the mean T1 weighted image. (**B**) Example of the left and right habenula regions manually identified on a participant's T1 weighted image. (**C**) Identification of the SN/VTA complex in a coronal (top) and axial (bottom) view. The SN/VTA was drawn using the mean proton density weighted MR image. (**D**) Identification of the PAG. The PAG was drawn using the mean T1 weighted image. SN/VTA, substantia nigra/ventral tegmental area; PAG, periaqueductal gray; leftHb, left habenula; rightHb, right habenula.

Images were acquired with a 3‐T TimTrio Siemens scanner at the Human Neuroimaging Laboratory‐Virginia Tech Carilion Research Institute (VTCRI). First, high‐resolution (0.5 × 0.5 × 1 mm) T1 weighted structural images were acquired with a MP‐RAGE pulse sequence. To set up the resting state functional images acquisition, the midbrain was identified on the central sagittal slice of the structural image and a slab comprising of 1.9‐mm thick coronal slices was centered on the midbrain and tilted to overlap as much as possible with the SN/VTA. Resting state functional images were acquired using a high‐resolution cardiac‐gated echo‐planar imaging (EPI) sequence (122 × 122 matrix, 1.5 × 1.5 mm^2^ in plane voxels, 1.9 mm thick slices, TE 43 ms, effective echo spacing 0.76 ms, partial Fourier factor = 7/8) (Fig. 1A; see Supporting Information Fig. 1 for the effective coverage of our coronal slab acquisition). For details about the cardiac gaited acquisition see SOM.

After the resting state functional scanning, a non‐cardiac‐gated whole‐brain functional image (TR/TE 2,650/43 ms, 25 6‐mm thick slices, FA 60°, four volumes, gap set individually so that all 25 coronal slices covered the brain) with the same center and orientation as the resting state functional images was acquired to facilitate the registration of the whole‐brain structural image to the functional data [D'Ardenne et al., 2008].

To facilitate the localization of the SN and VTA, a proton‐density weighted image (TR/TE 6,000/14 ms, FA 149°, echo spacing 14.1 ms, 0.75 × 0.75 mm^2^ voxels in plane, 20 1.9‐mm thick slices) was acquired using a turbo spin echo sequence with the slice center and orientation information from the functional images.

### MR Image Analysis

Preprocessing and fMRI analyses were performed using AFNI [Cox, 1996]. We corrected for the signal intensity variations caused by cardiac‐gated acquisition using a script written and implemented in MATLAB (The MathWorks, Natick, MA) based on the computation presented in Guimaraes et al. [1998]. Briefly, the longitudinal relaxation time constant, *T1e*, was estimated for each voxel based on the fact that the measured signal strength varies in proportion to the exponential recovery in longitudinal magnetization from one image acquisition to the next. Then

$$S_{i,n}=A_{i,n}{\left[1‐e^{({‐t}_{n}/{T1}_{i})}\right]}^{}$$

where $S_{i,n}\;is\;the\;measured\;signal\;for\;the\;ith\;voxel\;at\;the\;nth\;acquisition,\;t_{n}$ is the time between the n − 1 and nth image acquisitions, *T*1_i_ is the effective longitudinal relaxation time for the ith voxel, and *A*_i,n_ would be the maximum signal amplitude for the ith voxel at the nth image acquisition without the T1‐weighting. Note that $A_{i,n}=\;S_{i,n}{\left[1‐e^{\left({‐t}_{n}/{T1}_{i}\right)}\right]}^{‐1}$.

We estimated *T1e* as the time constant that minimized the variation in the measured signal for each voxel given the image acquisition times using coefficient of variation as our cost metric. We then computed the corrected fMRI signal (*Sc*) based on the estimated T1 (*T1e*) value for each voxel as:

$${Sc}_{i,n}=A_{i,n}\left[1‐e^{\left({‐T}_{av}/T1e\right)}\right]$$

where ${Sc}_{i,n}$ is the corrected signal for the ith voxel and the nth image acquisition, *T1e* is the estimated relaxation time constant and $T_{av}$ is average interacquisition interval given by $T_{av}=\left(\frac{1}{N}\right)\sum_{n=1}^{N}t_{n}$ where *N* is the total number of images and $t_{n}$ is the time between the n − 1 and nth image acquisitions. Then, functional images were despiked, corrected for slice‐timing offset and then for motion. Motion correction parameters were visually inspected and subjects with motion greater than 1.5 mm in any direction were removed from the analysis. Finally, the first and last coronal slice of each participant was removed because these may shift into unexcited regions.

Each participant's unnormalized structural images were segmented into white matter, gray matter, and cerebrospinal fluid using AFNI's segmentation tool 3dSeg. The white matter and cerebrospinal fluid masks were eroded by removing a one‐voxel layer. The structural image was aligned with the functional data using the whole‐brain non‐cardiac‐gated functional image [see Fig. 1 in Limbrick‐Oldfield et al., 2012 to visualize the method). The aligned structural image was normalized to Talairach space using afni's @auto_tlrc command (affine registration, linear interpolation) and then normalized into brainstem space using the technique developed by Napadow et al. [2008]. Finally, the proton‐density weighted images were also normalized to Talairach and brainstem spaces using the transform parameters from the structural normalization step.

### Habenula, SN/VTA, and Periaqueductal Gray Identification

Each participant's Hb was precisely identified and manually drawn on their unnormalized structural images using the procedure presented in [Lawson et al., 2013] (Fig. 1B). Left and right Hb volumes were calculated using SPM's Marsbar [Brett et al., 2002] toolbox. Since we were particularly interested in the connections between the Hb and structures in the midbrain, we defined regions of interest (ROIs) for the SN/VTA and the periaqueductal gray (PAG). The SN and VTA are easily localized on a proton‐density weighted image because the SN appears as high intensity bands between the peduncles and the circular red nucleus. The VTA can be identified as the region laying medially to the SN in the rostral two‐thirds of the midbrain. A group proton‐density weighted image was calculated by taking the mean from all the participants' proton‐density weighted images. The borders of SN and VTA were visually identified on this group image. Using AFNI's drawing tool, a single SN/VTA ROI was drawn for the entire group. The resulting SN/VTA ROI was comprised of 665 voxels (Fig. 1C). The PAG was manually identified and drawn on the mean group T1 weighted image. The PAG ROI was comprised of 248 voxels (Fig. 1D). ROIs were binarized in functional space using AFNI's 3dfractionize function with the subjects' functional image as the template and a clip value of 0.3 (i.e., clip off voxels that are <0.3 occupied). ROIs were individually inspected for possible errors that could have resulted from their interpolation/registration to the functional image's resolution (see Supporting Information Table 1 for data on the number of voxels within the left and right habenula for each participant).

### Preprocessing for the Functional Connectivity Analyses

Before functional connectivity analyses, fluctuations unlikely to be related to the specific regional correlations were removed. First, we computed the average EPI time series from voxels in (a) the white matter mask and, (b) the cerebrospinal fluid mask. Note that these EPI time series were extracted from the unsmoothed functional images in native space. Second, the average white matter and cerebrospinal fluid EPI time series in addition to the six movement parameters and a vector comprised of the duration of each TR were regressed out of the raw resting state functional signal. Next, the average time series from two seed regions was extracted: the left habenula, and the right habenula. In order to limit possible contamination by the bold signal from adjacent structures and possible problems arising from normalization [see Lawson et al., 2013], time series were extracted from unsmoothed functional images in native space. Finally, the “clean” unsmoothed functional images were smoothed using a 3 mm FWHM Gaussian kernel and normalized to Talairach and brainstem space using the same procedures presented earlier.

### Functional Connectivity Analyses

Functional connectivity was assessed at the subject level by computing the correlation coefficient between each of the Hb seed regions' mean signal (from unsmoothed data) and the “clean” smoothed normalized signal from every voxel in the acquired volume (coronal slab). Resulting correlation maps were transformed into Z scores by applying Fisher's r‐to‐Z transformation. Individual Z‐scored correlation maps were then entered in a second level random effect analysis to test the presence of correlation at the group level. All group results were FWE corrected based on AFNI's 3dClustSim function. The entire acquisition volume cluster activation FWE threshold was determined to be a minimum of 23 voxels at *P* < 0.005 (two‐tailed) uncorrected, corresponding to a *P* < 0.05 corrected threshold. Using the same approach, the FWE threshold was determined to be 6 voxels for the SN/VTA ROI and 5 voxels for the PAG ROI.

## RESULTS

### Hb Volumes

The average volume of the leftHb was 27.88 mm^3^ (SD = 8.49) while the average volume of the rightHb was 28.03 mm^3^ (SD = 8.18). leftHb and rightHb volumes were not statistically different (*t*(33) = −0.20, *P* > 0.05).

### Correlation Between leftHb and rightHb Seed Signals

We first assessed how similar the resting state signals from the leftHb and rightHb were. The mean Z‐scored correlation coefficient between the signal from the leftHb and the rightHb across participants was 0.06 (SD = 0.21) which was not statistically different from 0 (*t*(33) = 1.75, *P* > 0.05). We computed the scaled JZS (for Jeffreys, Zellner, and Siow) Bayes Factor [Rouder et al., 2009] for this one‐sample *t*‐test using a scale factor of 1 on the prior on effect size. The Bayes Factor allows the precise calculation of the preference for the null hypothesis or the alternative. We found a Bayes Factor of 1.79 in favor of the null hypothesis. We also conducted similar analyses on other control bilateral structures chosen from AFNI's TTatlas and within our coronal acquisition slab. These control ROIs were spheres of 2 mm radius (in order to have ROIs similar in size to the Hb) built around the coordinates given in the atlas. Data from these analyses show that the correlation between bilateral structures can span from absent to relatively high (Supporting Information Table 2). These results suggest that, at rest, the brain signals recorded from the left and right habenula are different.

### LeftHb and RightHb Functional Connectivity

**Figure 2:**
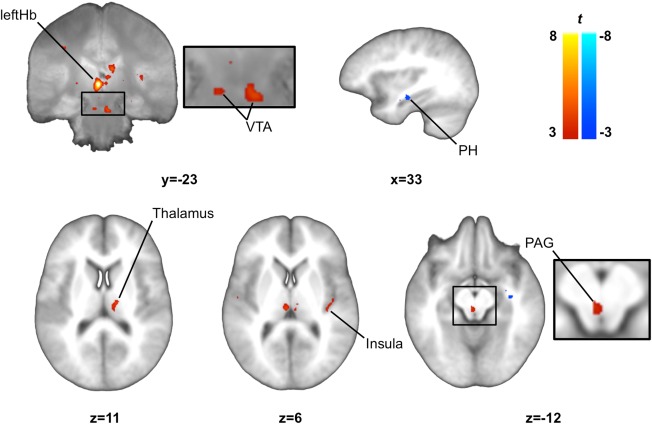
Functional connectivity map for the left habenula. We observed that the resting state activity in the left habenula showed a significant positive correlation with activity in the SN/VTA (VTA), thalamus, right posterior insula, PAG and a significant negative correlation with the activity in the parahippocampus. SN/VTA and thalamus clusters are displayed on the mean proton‐density weighted image while other clusters are displayed on the mean T1 weighted image. Images were thresholded at *P* < 0.005 uncorrected for display purposes. leftHb, left habenula; PAG, periaqueductal gray; PH, parahippocampus; SN/VTA, substantia nigra/ventral tegmental area.

Signal in the leftHb seed region was positively correlated with signal in the bilateral thalamus, right insula, PAG and in bilateral VTA: within regions of the middle ventral SN/VTA complex. Our analyses also showed that the signal in the leftHb was negatively correlated with a region of the left parahippocampus (Table 1; Fig. 2).

**Table 1:** Summary of the left and right habenula functional connectivity analyses

**Brain region**	**L/R**	**Voxels**	***t***	***x***	***y***	***z***
**Left Hb‐whole brain**						
Diencephalon	L	284	10.42	−2	−24	2
*Left Hb extending to left Thalamus*	L	*	10.92	−2	−24	1
*Thalamus*	R	*	5.13	−11	−16	15
SN/VTA (VTA)	R	46	4.20	4	−22	−20
Insula	R	40	3.68	37	−22	7
Parahippocampus	R	29	−4.86	35	−14	−12
**Left Hb‐ROI**						
PAG	L	19	3.83	−1	−26	−12
SN/VTA (VTA)	L	9	3.59	−6	−24	−22
**Right Hb‐whole brain**						
Diencephalon	R	309	12.75	2	−24	2
*Right Hb*	R	*	12.75	2	−24	2
*Thalamus*	L	*	5.16	−6	−22	7
*Thalamus*	R	*	4.99	7	−22	10
*Thalamus*	R	*	3.84	2	−14	1
*Thalamus*	L	*	3.73	−12	−27	10
Parahippocampus	R	27	3.62	24	−27	−14
Caudate body	L	25	−3.42	−18	−18	23
Pons	R	24	5.70	9	−27	−36
**Right Hb‐ROI**						
SN/VTA (SN)	R	14	3.89	10	−24	−18
SN/VTA (VTA)	R	7	−4.13	2	−22	−14
**Left Hb > Right Hb‐whole brain**						
Left Hb	L	36	8.58	−2	−24	2
**Left Hb > Right Hb‐ROI**						
SN/VTA (VTA)	R	11	3.92	2	−20	−15
**Right Hb > Left Hb‐whole brain**						
Right Hb	R	41	8.19	2	−24	1
Parahippocampus	R	25	3.57	37	−12	−15
**Right Hb > Left Hb‐ROI**						
SN/VTA (SN)	R	12	3.83	9	−24	−17

Clusters' volume, peak voxel coordinates in brainstem normalized Talairach space and *t* statistics. Whole‐brain results survived FWE *P* < 0.05 corrected, 23 voxels minimum cluster size. ROI results survived FWE *P* < 0.05 corrected, 6 voxels minimum cluster size for the SN/VTA ROI and 5 voxels minimum for the PAG ROI. *Italic font* indicates subpeaks within the cluster listed above.

L, left; R, right; PAG, periaqueductal gray; Hb, habenula; SN/VTA, substantia nigra/ventral tegmental area; ROI, region of interest.

**Figure 3:**
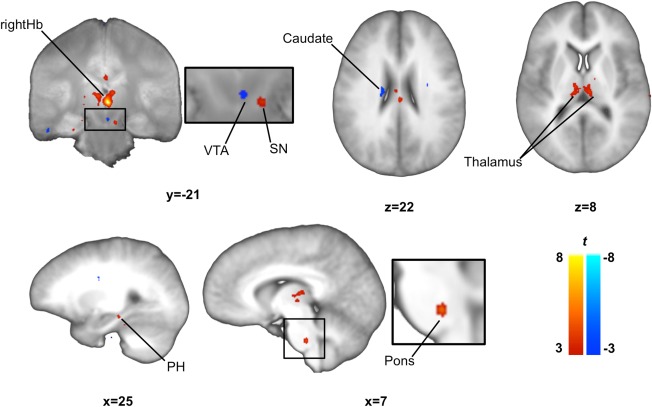
Functional connectivity map for the right habenula. We observed significant positive correlations between the resting state activity of the right habenula and activity in a cluster of the SN/VTA (SN), the parahippocampus, pons and thalamus and significant negative correlations with another cluster in the SN/VTA (VTA) and the caudate. SN/VTA clusters are displayed on mean proton‐density weighted image while other clusters are displayed on the mean T1 weighted image. Images were thresholded at *P* < 0.005 uncorrected for display purposes. rightHb, right habenula; PH, parahippocampus; SN/VTA, substantia nigra/ventral tegmental area.

Our functional connectivity analyses revealed a positive correlation between the signal in the rightHb and signal within the bilateral thalamus, a ventral region in the right pons and in the right SN. The rightHb was negatively correlated with signal in the left caudate body and within another cluster of the SN/VTA complex within the right VTA (Table 1; Fig. 3).

Finding regions of the SN/VTA coupled with the Hb prompted us to investigate the functional connections of these regions with further exploratory analyses. Because of the role of the habenula‐SN/VTA network in decision‐making, we were especially interested in their functional connections with regions of the reward/dopamine system and/or involved in goal‐directed actions. Using clusters within the SN/VTA as seed regions we found that the first cluster (Talairach‐brainstem space XYZ coordinates: 4 −22 −20) identified in the leftHb analysis was positively correlated with another region of the right dorsal SN/VTA, within the SN (42 voxels cluster, *P* < 0.05 FWE corrected) and negatively correlated with a region of the left caudate body (27 voxels cluster, *p* < 0.05 FWE corrected) (Supporting Information Fig. 2; bottom). No such functional connectivity was found for the second SN/VTA cluster (Talairach‐brainstem space XYZ coordinates: −6 −24 −22) identified in the leftHb analysis. The SN/VTA cluster (Talairach‐brainstem space XYZ coordinates: 2 −22 −14) negatively correlated with the rightHb was found to be positively correlated with a region of the caudate body (11 voxels cluster, uncorrected at *P* < 0.005) (Supporting Information Fig. 2; top) while no such functional connectivity was found for the cluster (Talairach‐brainstem space XYZ coordinates: 10 −4 −18) positively correlated with the rightHb.

### Differences Between LeftHb and RightHb Functional Connectivity

**Figure 4:**
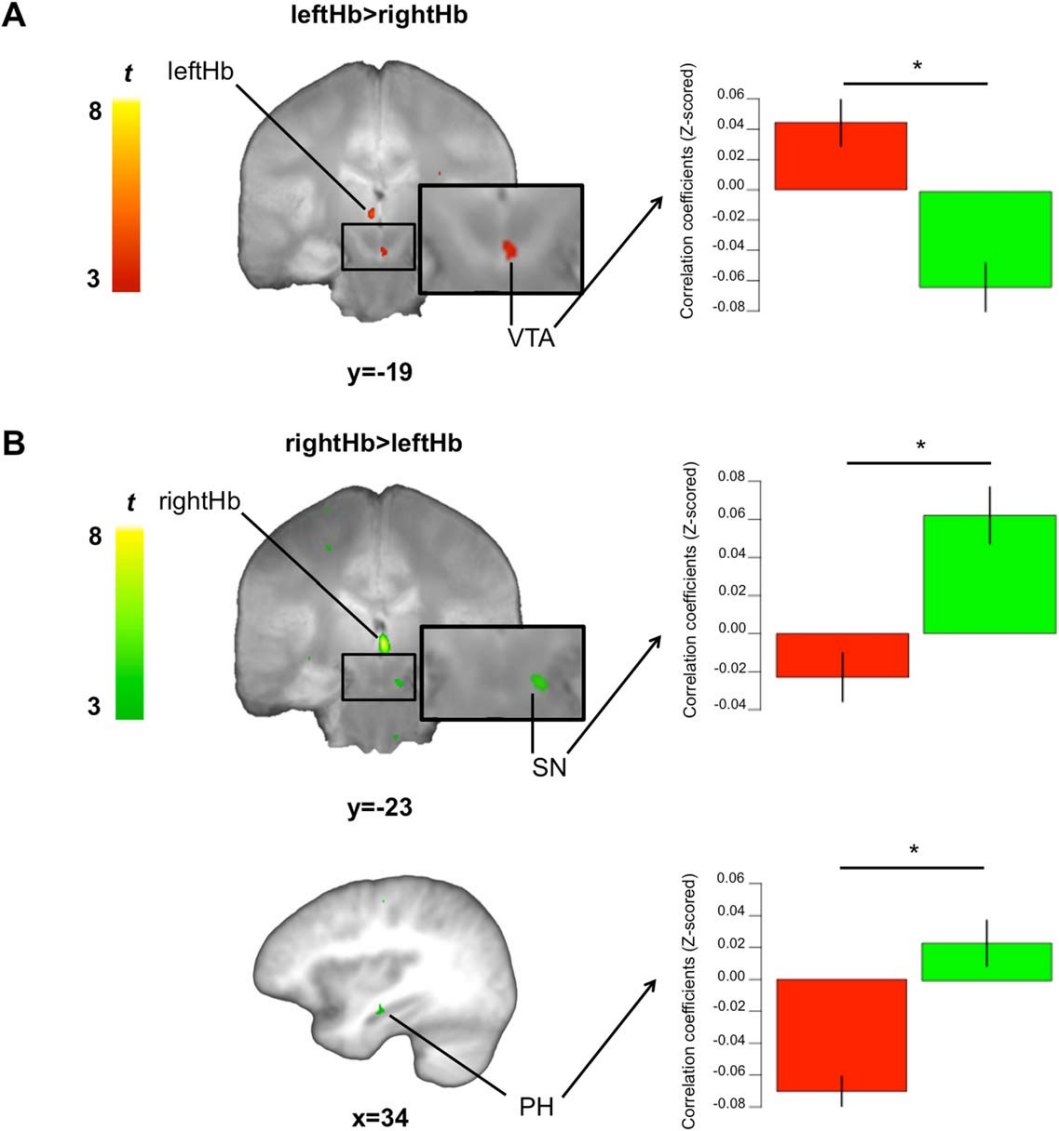
Differences in functional connectivity between the leftHb and rightHb. (**A**) Left: results of the contrast leftHb>rightHb. Right: We extracted each mean subject's Z‐scored correlation coefficients within the SN/VTA (VTA) cluster identified by this contrast. Average Z‐scored correlation coefficients for the leftHb (*red*) and rightHb (*green*) are presented in the bar graph. We showed that this SN/VTA cluster is more negatively correlated with the rightHb than the leftHb. (**B**) Left: results of the contrast rightHb>leftHb. Right: We extracted each mean subject's Z‐scored correlation coefficients within the SN/VTA (SN) and parahippocampus clusters identified by this contrast. Average Z‐scored correlation coefficients for the leftHb (*red*) and rightHb (*green*) are presented in the bar graph. We showed that the SN/VTA cluster is more positively correlated with the rightHb than the leftHb while the parahippocampus cluster is more negatively correlated with the leftHb than the rightHb. SN/VTA clusters are displayed on the mean proton‐density weighted image while the parahippocampus cluster is displayed on the mean T1 weighted image. Error bars represent SEM. rightHb, right habenula; leftHb, left habenula; PH, parahippocampus; SN/VTA, substantia nigra/ventral tegmental area; SEM, standard error of the mean. *P < 0.05 uncorrected.

Directly comparing the functional connectivity maps from the leftHb and rightHb (leftHb > rightHb and rightHb > leftHb) at the group level revealed a cluster within the right VTA where Z‐scored correlation coefficients were greater for leftHb than rightHb. To further investigate this difference, we extracted each subjects' mean Z‐scored correlation coefficients within the cluster identified from this contrast (leftHb > rightHb) and conducted additional exploratory ROI analyses. After removing outliers (±2SD from the group's mean Z‐scored correlation coefficient), this ROI analysis confirmed that Z‐scored correlation coefficient was higher for leftHb than rightHb (*t*(28) = 6.03, *P* < 0.001). Closer examination of this result revealed that this difference was due to the fact that this region was significantly negatively correlated with the rightHb (one sample *t*‐test: *t*(28) = −3.98, *P* < 0.001) while its functional connectivity with leftHb was significantly positively correlated with rightHB (one sample *t*‐test: *t*(28) = 2.85, *P* < 0.05) (Fig. 4). We also found a cluster in the right SN whose Z‐scored correlation coefficient was higher for the rightHb than the leftHb. Using a similar approach (but with the contrast (rightHb > leftHb)), we confirmed this result (*t*(29) = 4.71, *P* < 0.001) and showed that the positive correlation coefficient was significantly greater than 0 for rightHb (one sample *t*‐test: *t*(29) = 4.16, *P* < 0.001) while it was not different from 0 for the leftHb (one sample *t*‐test: *t*(29) = −1.81, *P* > 0.05) (Fig. 4). Finally, we found a cluster in the right parahippocampus that had higher Z‐scored correlation coefficient with the rightHb than leftHb. This was confirmed by our ROI analysis (*t*(29) = 5.26, *P* < 0.001). However, this difference can be better described by a significant negative coupling between the leftHb and the parahippocampus (one sample *t*‐test: *t*(29) = −7.52, *P* < 0.001) and a positive coupling not significantly different from 0 between the rightHb and the parahippocampus (one sample *t*‐test: *t*(29) = 1.66, *P* > 0.05) (Fig. 4). In summary, we found clusters in the SN/VTA that were more functionally connected (negative and positive correlations) with the rightHb than leftHb while functional connectivity with the parahippocampus was greater (negative correlation) for the leftHb than the rightHb.

## DISCUSSION

There is little known about the functional connectivity of the human habenula. Using high‐resolution resting state cardiac‐gated fMRI tailored for imaging small structures in the diencephalon, midbrain and the brainstem, we report functional connections between the Hb and regions involved in pain and reward processing, highlighting the role the Hb as an important relay between the cortex and subcortical regions [Bianco and Wilson, 2009; Hikosaka, 2010; Lecca et al., 2014; Proulx et al., 2014]. Interestingly, our data also suggests differences in resting state functional connectivity between the left and right habenula.

### Functional Connectivity of the Habenula in Humans at Rest

When considering the data from both the left and right Hb, our results indicate that the Hb is functionally connected at rest with several cortical and subcortical regions. At the cortical level, the Hb was functionally coupled to the insula. At the subcortical level, the thalamus, striatum (caudate body), pons, SN/VTA complex, PAG, and limbic system (parahippocampus) were functionally connected with the Hb. Our results overlap with some of the known Hb afferents such as the VTA, and efferents such as the SN, VTA, and the thalamus that were experimentally identified in non‐human species [reviewed in Bianco and Wilson, 2009]. Our findings are also in agreement with a previous resting state study in a pediatric human sample that identified functional connections between the Hb and the insula, striatum, parahippocampus, and regions in the midbrain including the SN, VTA, and PAG [Erpelding et al., 2014]. Furthermore, a recent DTI study has revealed anatomical connections between the Hb and the SN/VTA and between the Hb and the PAG [Shelton et al., 2012b], two structures that were identified by our resting state functional connectivity analyses. It is important to note that with our cardiac‐gated high‐resolution approach we could not image the whole brain and thus, our results are limited to structures within a restricted volume. It is therefore possible that other regions of the brain outside our volume are also functionally connected to the Hb. Indeed, using an approach where the volume of acquisition included the entire brain (but at a lower resolution; with the habenula seed region being only 2 voxels), a previous resting state study also provided evidence that the Hb was functionally connected with the cerebellum, primary and premotor motor cortices, primary and secondary somatosensory cortex, frontal pole, dorsolateral prefrontal cortex, anterior and middle cingulate cortex, and the precuneus [Erpelding et al., 2014]. Future studies using high‐resolution whole brain imaging will probably help draw a more complete and precise map of the Hb connectivity in humans. Nevertheless, our study provides some of the first data on the Hb resting state functional connectivity when this structure is precisely defined at the individual level.

Functional coupling between the Hb and cortical as well as subcortical regions is in agreement with the proposed role of the Hb as a relay between cognitive and sensory processing structures and the aminergic regions of the midbrain involved in affective and reward processing [Bianco and Wilson, 2009; Hikosaka, 2010; Proulx et al., 2014]. Indeed, our analyses revealed that regions known to be involved in pain and emotion processing, such as the posterior insula [Garcia‐Larrea and Peyron, 2013], the PAG [Linnman et al., 2012], and the limbic system [Frank et al., 2014; Lindquist et al., 2012], were functionally connected with the Hb. Moreover, several animal studies have shown that the Hb plays an important role in the processing of aversive stimuli [Cohen et al., 2012; Lammel et al., 2011, 2012; Matsumoto and Hikosaka, 2007, 2009; Stamatakis and Stuber, 2012]. Recent fMRI studies have extended these findings showing that the Hb is also involved in processing cues related to noxious stimuli in humans [Hennigan et al., 2015; Lawson et al., 2014]. Both of these studies also looked at the habenula's functional connectivity during aversive stimuli processing. Hennigan et al. [2015] showed that the functional connection between the VTA and the Hb (left Hb) was increased for cues predicting noxious compared with neutral stimuli while Lawson et al. [2014] showed that the Hb (right Hb) was functionally connected with several structures including the limbic system (amygdala) when considering the changing values of a noxious conditioning stimulus. This suggests that the functional connections we identified between the Hb and the VTA, the limbic system and the insula during rest could be part of the network signaling and processing aversive stimuli. For example, results from a recent study suggest that the amygdala's influence on the activity of the VTA is mediated by the Hb [Ide and Li, 2011]. One intriguing result from our study is that we found the parahippocampal signal to be negatively correlated with the signal from the Hb (left Hb). Previous studies have, on the contrary, found positive relations between the signal in the Hb and the signal in limbic structures [Erpelding et al., 2014; Ide and Li, 2011; Lawson et al., 2014]. This discrepancy may be partially due to the fact that Ide and Li [2011] and Lawson et al. [2014] measured functional connectivity not at rest but during error and noxious stimuli processing respectively. Thus, it is possible that the strength and direction of the connections between the limbic system and the Hb are modified during the processing of aversive stimuli compared with rest.

We also found that the Hb was functionally connected with the striatum, which is in line with the Hb role in reward and goal‐directed actions [Bianco and Wilson, 2009; Hikosaka, 2010; Proulx et al., 2014]. Furthermore, our high‐resolution imaging approach enabled us to confirm the functional connections between the Hb and the SN/VTA complex in humans, another important structure of the reward and learning system [Montague et al., 1996; Schultz, 2007a, 2007b; Schultz et al., 1997]. Based on brain atlases [Naidich et al., 2009], probabilistic maps [Murty et al., 2014], and high resolution data of the midbrain [Eapen et al., 2011], three regions identified by our analysis seemed to be located in the medial ventral section of the SN/VTA complex (Talairach‐brainstem space XYZ coordinates: 4 −22 −20; −6 −24 −22; 2 −22 −14) where the VTA lies, while the fourth cluster seemed to be located within the SN (Talairach‐brainstem space XYZ coordinates: 10 −24 −18). Using a similar high‐resolution fMRI approach but looking at connectivity during cued noxious stimuli, Hennigan et al. [2015] also identified a functional connection between the Hb (leftHb) and the VTA, although in a region more dorsal and lateral to ours. Animal studies have shown that the habenula plays a major role in controlling the activity of dopamine neurons in the SNcompacta and the VTA [Matsumoto and Hikosaka, 2007, 2009]. More precisely, the firing rate of Hb neurons increases during unexpected aversive stimuli and worse than expected rewards [Matsumoto and Hikosaka, 2007, 2009], while the firing rate of dopamine neurons increases in response to unexpected (better than expected) rewards [Fiorillo, 2013; Schultz, 2013; Schultz et al., 1997]. The fact that both structures are known to be part of the same circuit but exhibit opposite firing patterns hinted to the presence of an intermediate GABAergic structure that could translate activation/inhibition in the Hb into inhibition/activation in the SN/VTA. The rostromedial tegmental nucleus (RMTg), (also called the tail of the VTA [tVTA]), a region rich in GABAergic neurons which is located in the caudal section of the VTA, has been proposed to play this mediating role [reviewed in Barrot et al., 2012; Bourdy and Barrot, 2012; Lavezzi and Zahm, 2011]. Animal studies confirmed that the RMTg/tVTA received incoming excitatory signals from the Hb and projected inhibitory signals to the dopamine neurons in the SN/VTA [Hong et al., 2011; Jhou et al., 2009; Kaufling et al., 2009; Stamatakis and Stuber, 2012]. Interestingly, we found a cluster within the VTA that was negatively correlated with the signal in the rightHb suggesting a similar network in humans. Furthermore, using this VTA cluster as a seed, we found a region within the caudate body positively correlated with the signal in the VTA. This cluster in the caudate partially overlapped with the cluster negatively correlated with the rightHb. Taken together, these results could indicate that in humans, as in other animals [see Bianco and Wilson, 2009; Hikosaka, 2010; Proulx et al., 2014], there is a functional network where the habenula exerts an inhibitory influence on the dopamine system (possibly via the RMTg/tVTA) which in turn reduces the activity within the striatum. This interpretation has to be considered with care, as more work is clearly needed to first replicate our results and second directly test this hypothesis.

Even if ultimately, the existence of RMTg/tVTA in human needs to be validated in post‐mortem human brain tissue samples, we found that within two other regions of the VTA, the BOLD signal was positively correlated with the activity of the leftHb, which raises the possibility that these clusters could actually be part of the RMTg/tVTA. If this was the case, the “Hb to RMTg/tVTA to SN/VTA” network identified in rodents and non‐human primates would predict that signals within these clusters would be negatively correlated with other regions within the SN/VTA. Using these clusters as seed regions, we only found a positively correlated cluster within the SN for one of the cluster (Talairach‐brainstem space XYZ coordinates: 4 −22 −20) suggesting that these VTA clusters are not part of the RMTg/tVTA. It is important to note that since BOLD signal is only an imprecise and indirect measure of neuronal activity [Logothetis et al., 2001], we need to be cautious when interpreting positive/negative correlations as excitatory/inhibitory influences. This being said, an alternative hypothesis is that our analysis revealed direct connections between the leftHb and the VTA. Recent results have shown that the Hb also sends direct excitatory projections to the VTA [Gonçalves et al., 2012; Lammel et al., 2011, 2012]. Indeed, data from animal [Bromberg‐Martin et al., 2010; Cohen et al., 2012] and human [Hennigan et al., 2015] studies have shown that activity within the VTA can also increase when processing aversive stimuli. Furthermore, the rodent VTA also harbors a subpopulation of GABAergic neurons that receive direct inputs from the Hb and project back to it [Stamatakis et al., 2013]. Because of the important heterogeneity within the VTA [Liss and Roeper, 2008; Sanchez‐Catalan et al., 2014; Walsh and Han, 2014], it is currently impossible to precisely identify which (if any) subpopulation of neurons within the VTA is functionally connected with the Hb in humans using fMRI. Furthermore, identification and localization of the RMTg/tVTA remain difficult even in rodents, where invasive techniques are available [Lavezzi and Zahm, 2011]. Therefore, if our results provide strong evidence that the Hb is functionally coupled to the VTA in humans at rest, they unfortunately cannot inform us on the exact nature of these connections. Development in higher fields [Eapen et al., 2011] and faster acquisition [Zahneisen et al., 2014], resulting in higher‐resolution imaging in addition to the use of experimental protocols and causal modeling analyses [Stephan and Roebroeck, 2012], could potentially help overcome this challenge. This could also help better define the nature of the connection between the Hb and the SN/VTA identified by our analysis.

### Laterality Differences in the Habenula Functional Connectivity

Interestingly, while our data confirmed previous results from in‐vivo [Lawson et al., 2013] and post‐mortem [Ranft et al., 2010] studies showing that morphologically, the leftHb and rightHb do not differ in humans, our study also revealed a possible laterality asymmetry in the functional connectivity of the habenula in humans at rest. The first evidence of this is the fact that the resting state activity recorded from the left and right Hb does not seem to be correlated. We still want to point out that even if we statistically confirmed using the Bayes Factor that the mean time series from the left and right habenula were different, the evidence for this is not as strong as for other structures. Second, except for the coupling with the bilateral thalamus and the parahippocampus, qualitatively the functional connectivity maps from the leftHb and rightHb differed greatly. Furthermore, even if our data suggests that both leftHb and rightHb are functionally coupled with the parahippocampus, this coupling is in opposite direction (leftHb: negative correlation; rightHb: positive correlation). Third and most importantly, when directly comparing the functional connectivity maps from the leftHb and rightHb, we found increased coupling with the rightHb (in terms of positive and negative correlations) in regions of the SN/VTA and greater coupling between the leftHb (negative correlation) and the right parahippocampus. Functional, morphological and structural differences between the right and left Hb have been studied extensively in phylogenetically older species [Bianco and Wilson, 2009; Villalón et al., 2012]. Laterality differences have even been shown to be necessary for proper sensory processing in the zebra fish [Dreosti et al., 2014]. How these laterality differences are expressed seems to be highly species dependent [Bianco and Wilson, 2009; Concha and Wilson, 2001], which makes any inference about humans difficult. Furthermore, previous work that has looked at the connectivity of the human Hb has often considered the left and right Hb as a single structure [Ide and Li, 2011; Shelton et al., 2012b]. Nevertheless, others looking at the Hb function in processing noxious stimuli have separately investigated each side but found conflicting results [Hennigan et al., 2015; Lawson et al., 2014]. On one hand, Lawson et al. [2014] found that only the rightHb coded for the trial‐by‐trial variations in value for cues predicting foot shocks. The authors suggested that this laterality difference probably resulted from the fact that they were applying the shocks to the left side of the body. On the other hand, Hennigan et al. [2015] using shocks on the left foot, found higher reactivity to cues predicting noxious stimuli versus neutral cues only in the leftHb. Unfortunately, the only other study that looked at the Hb functional connectivity at rest did not directly compare leftHb versus rightHb coupling even though they present separate results for the two sides [Erpelding et al., 2014]. Qualitatively, similar to our results, their data suggests that the leftHb and rightHb have different functional connectivity maps. Still, since Erpelding et al. [2014] looked at connections throughout the entire brain but used a lower‐resolution imaging approach in a pediatric sample, one must be cautious when trying to directly compare their results to ours. Taking into consideration these previous research and the data from the present study, it is clear that more work is needed to (1) test possible laterality differences in the functions and connections of the Hb in humans and (2) investigate if and how these differences are modulated by going from rest to tasks recruiting the Hb. Indeed, while work on habenular laterality differences in other species such as the zebra fish have produced important results describing these differences [Hong et al., 2013] and the mechanism involved in laterality differentiation [Concha et al., 2009; Hüsken et al., 2014], data in humans and non‐human primates remain sparse at best.

### Limitations

When looking at our results, one should take into consideration some limitations. We used a state of the art high‐resolution cardiac‐gated imaging approach; however, this came at a cost, we could only look at the coupling between the Hb and structures that lied within a coronal slab centered over the brainstem (see Fig. 1C). Therefore, we could not report on Hb connections with structures in the frontal cortex or basal ganglia that have been previously identified in animal studies [Shabel et al., 2012]. Second, it is known that the Hb can be further divided into the medial and lateral nuclei that have different inputs/outputs and different functions [Lecca et al., 2014; Viswanath et al., 2014]. Unfortunately, it is impossible to precisely differentiate the lateral Hb and medial Hb with the current method used to identify the human Hb on a structural image [Lawson et al., 2013]. Finally, even if our habenula volumes are very similar to previously published in‐vivo results from healthy adults [Lawson et al., 2013] and post‐mortem data [Ranft et al., 2010], they are larger than other in‐vivo results [Savitz et al., 2011a, 2011b]. This discrepancy is probably due to the fact that we used the technique presented in Lawson et al., 2013 which is based on but different than the one used in Savitz et al., 2011a,b. Note that we opted for this approach because it is the one that was used in recently published articles looking at the habenula's function in humans [Hennigan et al., 2015; Lawson et al., 2014].

## CONCLUSION

The habenula has been proposed to play a role in mood disorders and addiction [Bourdy and Barrot, 2012; Hikosaka, 2010; Lammel et al., 2014; Lecca et al., 2014; Proulx et al., 2014], but its functional connectivity remains mostly underexplored in humans. Our study presents the first results on the Hb resting state functional connectivity using a high‐resolution cardiac‐gated imaging approach optimized for small structures in the brainstem/midbrain/diencephalon. This enabled us to identify several connections between the Hb and cortical and small subcortical structures that had previously been shown in non‐human animal studies, confirming the role of the Hb as a hub for cognitive and emotional signals to the aminergic centers of the brain. Furthermore, our study presents evidence for a lateral asymmetry in the resting state functional connectivity of the Hb with stronger coupling between the rightHb and the SN/VTA and stronger coupling between the leftHb and the limbic system. As the Hb is increasingly considered as a potential target for treatment of psychiatric disorders [Cleary et al., 2015; Sartorius and Henn, 2007], our results provide much needed data on the functional coupling of this structure in humans.

### Som

#### Cardiac gaiting acquisition

A pulse‐oxymeter interfacing with the MR scanner was placed on the middle finger of the participant's non dominant hand to monitor his pulse. During the anatomical scan, the experimenter measured the participant's heart rate and used it to determine the number of slices for the functional scan. The number of slices remained constant throughout the experiment. The heart rate was also used to select the volume acquisition time (TR in non‐cardiac‐gated imaging) and the maximum length of the acquisition window. The acquisition window is the amount of time the scanner will wait before launching another volume acquisition. The scanner was set to acquire an image every second heartbeat. For a subject with a heart rate of 60 beats per minute, 18 slices would be used, with a volume acquisition time of 1,910 ms and an acquisition window of 1,960 ms. The volume acquisition time was always set to be as fast as possible, depending on the number of slices used. The flip angle (FA) was calculated according to the Ernst angle:

$$\cos\left(\alpha_{E}\right)=e^{\left(‐\frac{TR}{T1}\right)}$$

The flip angle is $\alpha_{E}$, TR is the repetition time or acquisition time, and T1 is the T1 value for gray matter at 3T (i.e., 1,325 ms; Zhang et al., 2006 **)**. For 8–9 slices, FA = 60°; for 10–13 slices, FA = 70°; for 14–15 slices, FA = 75°; and for 16–20 slices, FA = 80°.

The mean number of slices was 14.6 ± 2.3 (SD) (range = 11–18) while the mean volume acquisition time (TR) was 1,547.9 ms ± 249.7, and the mean acquisition window was 1,597.9 ms ± 249.7.

## Supporting information

Supporting Information Figure 1

Supporting Information Figure 2

Supporting Information Table 1

Supporting Information Table 2
