# Supplementary figures and images for: Asymmetry in functional connectivity of the human habenula revealed by high‐resolution cardiac‐gated resting state imaging
Source: Hum Brain Mapp. 2016 Apr 1;37(7):2602–15. doi: 10.1002/hbm.23194 (PMC4905773; doi:10.1002/hbm.23194)

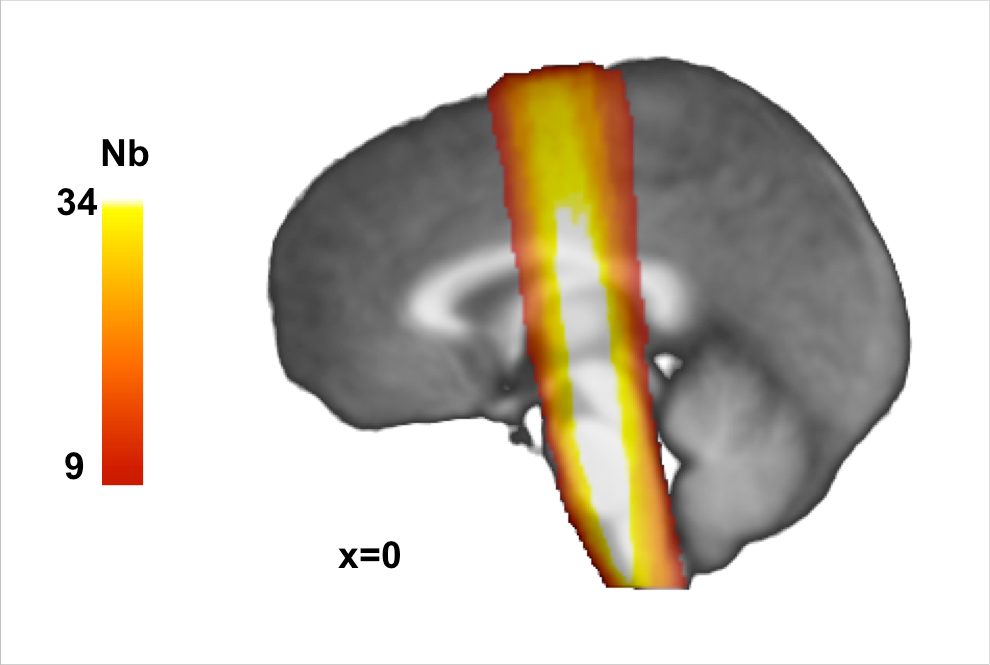

Supplement: Supplementary file 1 — Supporting Information Figure 1 [file HBM-37-2602-s001.tif]

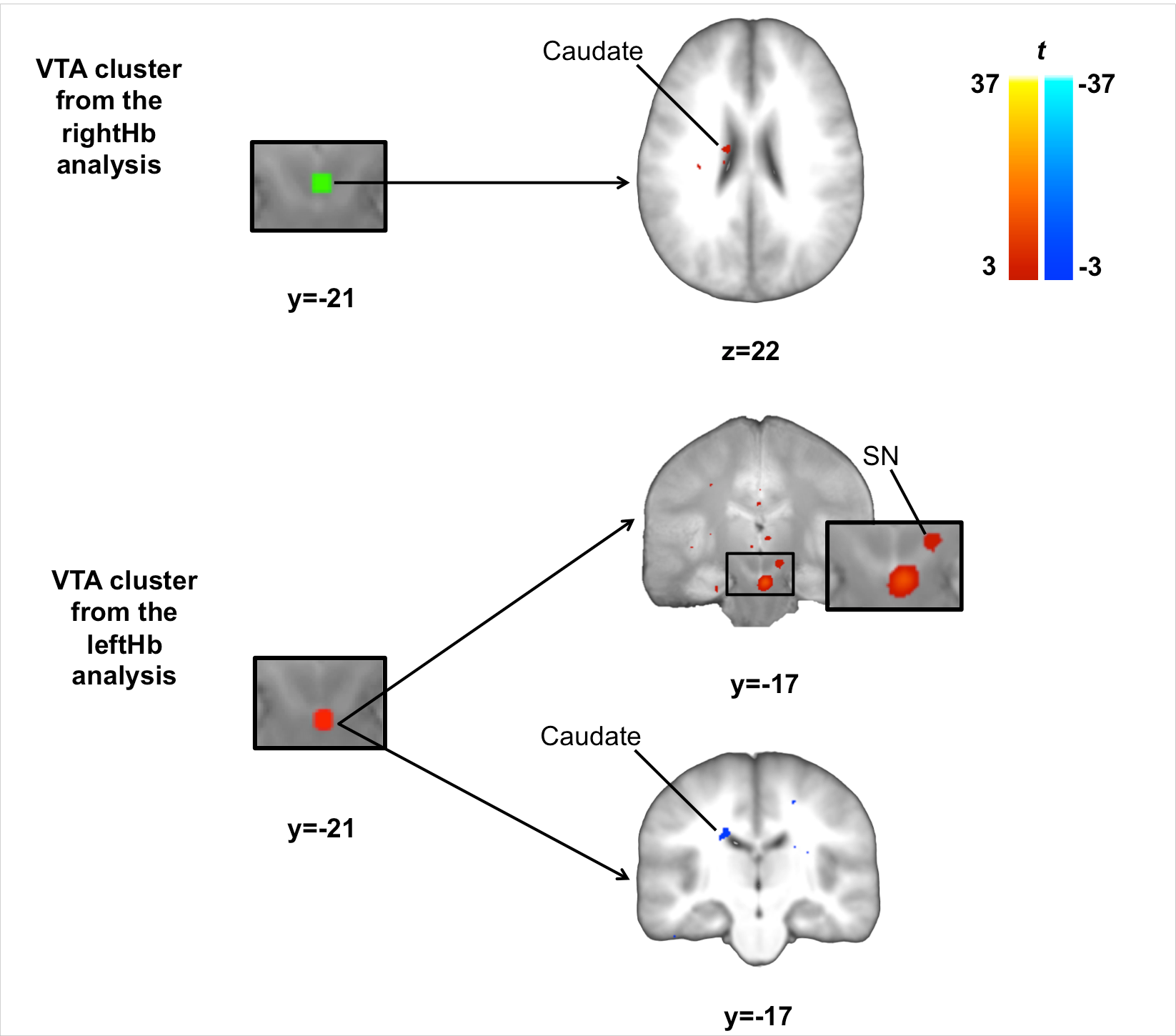

Supplement: Supplementary file 2 — Supporting Information Figure 2 [file HBM-37-2602-s002.tif]
