# Supplementary material for: Asymmetry in functional connectivity of the human habenula revealed by high‐resolution cardiac‐gated resting state imaging
Source: Hum Brain Mapp. 2016 Apr 1;37(7):2602–15. doi: 10.1002/hbm.23194 (PMC4905773; doi:10.1002/hbm.23194)
Supplement: Supplementary file 3 — Supporting Information Table 1 [file HBM-37-2602-s003.docx]

Supplementary table 1. Number of voxels in left and right habenula masks

| Subject # | Number of voxels in left habenula | Number of voxels in right habenula |
| --- | --- | --- |
| 1 | 7 | 7 |
| 2 | 8 | 12 |
| 3 | 6 | 5 |
| 4 | 8 | 8 |
| 5 | 7 | 7 |
| 6 | 11 | 6 |
| 7 | 10 | 8 |
| 8 | 6 | 11 |
| 9 | 7 | 8 |
| 10 | 8 | 5 |
| 11 | 8 | 8 |
| 12 | 5 | 8 |
| 13 | 11 | 11 |
| 14 | 9 | 9 |
| 15 | 7 | 7 |
| 16 | 5 | 5 |
| 17 | 10 | 10 |
| 18 | 10 | 10 |
| 19 | 6 | 8 |
| 20 | 5 | 4 |
| 21 | 8 | 8 |
| 22 | 11 | 8 |
| 23 | 9 | 6 |
| 24 | 6 | 7 |
| 25 | 5 | 7 |
| 26 | 7 | 5 |
| 27 | 7 | 7 |
| 28 | 6 | 9 |
| 29 | 6 | 9 |
| 30 | 8 | 7 |
| 31 | 7 | 7 |
| 32 | 10 | 9 |
| 33 | 10 | 11 |
| 34 | 5 | 5 |

Number of voxels in the left and right habenula masks for each participant after interpolation/registration with the functional images.
