# Supplementary material for: Asymmetry in functional connectivity of the human habenula revealed by high‐resolution cardiac‐gated resting state imaging
Source: Hum Brain Mapp. 2016 Apr 1;37(7):2602–15. doi: 10.1002/hbm.23194 (PMC4905773; doi:10.1002/hbm.23194)
Supplement: Supplementary file 4 — Supporting Information Table 2 [file HBM-37-2602-s004.docx]

Supplementary table 2. Correlation between left and right structures’ mean BOLD time series

| ROI name | z-scored correlation coefficient  Mean (SD) | One sample *t*-test result  t(df), p value | Bayes Factor  (in favor of null or alternative) |
| --- | --- | --- | --- |
| Thalamus | 0.01 (0.13) | 0.48(28), .64 | 6.23 (null) |
| BA 41(primary auditory cortex) | 0.02 (0.14) | 0.50(22), .62 | 5.54 (null) |
| ***BA 41(primary auditory cortex)*** | ***0.30 (0.25)*** | ***6.77(32), <.01*** | ***1.38E5 (alternative)*** |
| Pulvinar | 0.02 (0.16) | 0.50(21), .62 | 5.43 (null) |
| Medial globus pallidus | -0.01 (0.13) | -0.47(17), .64 | 5.03 (null) |
| BA 22 (supplementary temporal gyrus) | 0.03 (0.14) | 1.23(26), .23 | 3.30 (null) |
| BA 35 (perirhinal cortex) | 0.04 (0.16) | 1.42(31), .17 | 2.81 (null) |
| Parrahippocampal gyrus | 0.04 (0.15) | 1.51(31), .14 | 2.50 (null) |
| Lateral globus pallidus | -0.04 (0.11) | -1.59(18), .13 | 1.83 (null) |
| BA4 (primary motor cortex) | 0.04 (0.12) | 1.66(24), .11 | 1.83 (null) |
| ***BA4 (primary motor cortex)*** | ***0,19 (0.24)*** | ***4.30(29), <.01*** | ***147.99 (alternative)*** |
| **Habenula** | **0.06 (0.21)** | **1.75(33), .09** | **1.79 (null)** |
| BA 42(primary auditory cortex) | 0.06 (0.16) | 1.81(23), .08 | 1.44 (null) |
| Transverse temporal gyrus | 0.04 (0.13) | 1.93(31), .06 | 1.31 (null) |
| BA 21 | 0.08 (0.16) | 2.97(33), <.01 | 6.04 (alternative) |
| BA 6 (supplementary motor area) | 0.11 (0.15) | 3.54(20), <.01 | 18.13 (alternative) |
| BA 43 (primary gustatory cortex) | 0.15 (0.17) | 4.86(28), <.01 | 548.63 (alternative) |

The group’s mean z-scored correlation coefficients between the time series from various left and right ROIs. Results on the one sample *t*-test testing if the group’s mean correlation is different from zero and the associated scaled JZS Bayes Factor are also presented. The table is organized from the region with the most evidence for the null to the region with the most evidence for the alternative. ROIs were spheres of 2 mm radius around the coordinates from AFNI’s TTatlas except for the habenula ROI which were individually defined in each participants. **Since previous studies using larger ROIs had shown that the left and right sides of the primary auditory and motor cortices were functionally connected, we provide in italic results from larger ROIs (spheres of 6 mm radius) for these two structures.** Time series were extracted from the unsmoothed functional images and averaged over all the voxels inside the ROIs.

ROI: region of interest; SD: standard deviation; df: degrees of freedom; BA: Brodmann area.
